# Development and usability testing of an online platform for provider training and implementation of cognitive-behavioral therapy guided self-help for eating disorders

**DOI:** 10.21203/rs.3.rs-4409969/v1

**Published:** 2024-05-27

**Authors:** Laura D’Adamo, Agatha Laboe, Jake Goldberg, Carli Howe, Molly Fennig, Bianca DePietro, Marie-Laure Firebaugh, Zafra Cooper, Denise Wilfley, Ellen Fitzsimmons-Craft

**Affiliations:** Drexel University; University of Wisconsin–Madison; Washington University in St. Louis; Washington University in St. Louis; Washington University in St. Louis; Washington University in St. Louis; Washington University in St. Louis; Yale University; Washington University in St. Louis; Washington University in St. Louis

**Keywords:** eating disorders, cognitive-behavioral therapy, provider training, mental health treatment, dissemination and implementation, evidence-based treatment

## Abstract

**Background:**

Most individuals with eating disorders (EDs) do not receive treatment, and those who do receive care typically do not receive evidence-based treatment, partly due to lack of accessible provider training. This study developed a novel “all-in-one” online platform for disseminating training for mental health providers in cognitive-behavioral therapy guided self-help (CBTgsh) for EDs and supporting its implementation. The aim of the study was to obtain usability data from the online platform prior to evaluating its effects on provider training outcomes and patient ED symptom outcomes in an open pilot trial.

**Methods:**

Nine mental health provider participants (n = 4 in Cycle 1; n = 5 in Cycle 2) and 9 patient participants (n = 4 in Cycle 1; n = 5 in Cycle 2) were enrolled over two cycles of usability testing. In Cycle 1, we recruited providers and patients separately to complete brief platform testing sessions. In Cycle 2, we recruited provider-patient dyads; providers completed training using the platform and subsequently delivered CBTgsh to a patient for three weeks. Usability was assessed using the System Usability Scale (SUS), the Usefulness, Satisfaction, and Ease of Use Questionnaire (USE), and semi-structured interviews.

**Results:**

Interview feedback converged on two themes for providers (applicability of program for real-world use, platform structure and function) and two themes for patients (barriers and facilitators to engagement, perceived treatment effects). SUS and USE scores were in the “average” to “good” ranges across cycles.

**Conclusions:**

Findings from this study demonstrate preliminary feasibility and acceptability of the online platform. Data collected in this study will inform further refinements to the online platform. The platform’s effects on provider training outcomes and patient ED symptom outcomes will be evaluated in an open pilot trial. Given the wide treatment gap for EDs and barriers to dissemination and implementation of evidence-based treatments, the online platform represents a scalable solution that could improve access to evidence-based care for EDs.

## Background

Eating disorders (EDs) are serious mental illnesses that affect 10% of people in their lifetime (1). EDs are associated with high medical and psychiatric comorbidity, poor quality of life, and high mortality (2). Evidence-based treatments for EDs have been well-established (3, 4) and are recommended by treatment guidelines (5). Yet, less than 20% of those with EDs receive treatment (6, 7), and when individuals with EDs do receive care, it is typically not an evidence-based treatment (8–10). Further, some research has shown that providers working in certain settings, including community mental health clinics and rural areas, may be even less likely to use evidence-based protocols (11–13). Lack of accessible provider training in evidence-based treatments has been cited as a major contributor to the research-practice gap (14, 15). Standard methods for provider training, which typically consist of a one- or two-day workshop delivered by an expert and provision of a manual (16), require substantial time and resources, making dissemination difficult (9). This approach also is not sustainable, and as providers leave the site and new ones enter, new providers do not have access to training. Further, although workshops increase knowledge, their impact on skills is short-lived without ongoing support (16). Scalable and sustainable methods for provider training in evidence-based treatments for EDs and ongoing support are needed.

Online platforms can overcome barriers to dissemination and implementation of training (17) and have several advantages over traditional methods of training: 1) training can be offered to geographically dispersed providers; 2) training is accessible anytime, anywhere; 3) providers can repeatedly review material, reinforcing learning; 4) the platform can be regularly updated; 5) data on most-used features can be collected, informing refinement; and 6) online training is a sustainable resource to address the issue of turnover (18–20). Websites for evidence-based treatment training are starting to be developed (e.g., for motivational interviewing, cognitive-behavioral therapy, interpersonal psychotherapy, dialectical behavior therapy), have demonstrated efficacy (e.g., 21–27), and hold promise for training providers in rural areas (28). Although research has found that ongoing support is needed to sustain the impacts of training (16), providing traditional ongoing expert support can increase training costs by 50%+ (29). Electronic support tools may be a scalable solution for providing ongoing support and enhancing treatment implementation in several ways. First, checklists can help providers ensure essential components are delivered (30). Second, routine outcome monitoring, including electronic feedback systems, improves patient outcomes (31–33). Finally, electronic support tools can enhance homework compliance and facilitate information transfer to providers (34).

When selecting an evidence-based treatment to disseminate, it is important to consider efficacy; cost-effectiveness; clinical range; ease of training/learning; and mode of treatment delivery (e.g., with limited external input and by providers with minimal training) (9). Further, the difficulties that are often encountered when attempting to scale-up evidence-based treatments may be exacerbated by design problems, which may be addressed by user-centered design (also known as human-centered design or design thinking) (35). User-centered design is an approach to product development that grounds the process in information about the individuals and settings with which products will ultimately be used (35).

With target user input, this study developed a novel “all-in-one” online platform for both training mental health providers in an evidence-based treatment for EDs and supporting its implementation. Cognitive-behavioral therapy guided self-help (CBTgsh) was selected as the treatment of focus for several reasons. First, CBTgsh is effective in treating adults with bulimia nervosa (BN) and binge-eating disorder (BED) (36) and is recommended by the National Institute for Health and Care Excellence (NICE) as a first-line treatment for adults with BN and BED (37). Second, CBTgsh is acceptable to patients, cost-effective, requires less training than standard approaches, and is intended to be implemented by a wide variety of providers, including non-specialists (36). Finally, given CBTgsh’s self-help format, all patient-facing self-help content can be built into the online platform, creating a “one-stop shop” for providers and patients. This paper describes the process of developing the online platform and conducting two iterative cycles of usability testing with mental health providers without prior training in CBTgsh and adult patients with EDs. The aim of the study was to garner feasibility and acceptability data on the online platform prior to evaluating its effects on provider training outcomes and patient ED symptom outcomes in an open pilot trial.

## Methods

### Participants

Nine mental health provider participants (n = 4 in Cycle 1; n = 5 in Cycle 2) and nine patient participants (n = 4 in Cycle 1; n = 5 in Cycle 2) were enrolled over two cycles of usability testing. In Cycle 1, eligibility criteria for providers included: 1) 18 years or older, 2) mental health provider, 3) US resident, 4) English-speaking, and 5) no experience in providing CBTgsh (experience treating EDs with other modalities was permitted but not required or assessed). In Cycle 2, eligibility criteria were identical to those in Cycle 1, with the addition that eligible providers had to currently have or anticipate having a patient with an ED in the next two months. In Cycle 1, inclusion criteria for patients included: 1) 18 years or older, 2) US resident, 3) English-speaking, and 4) screening positive for clinical/subclinical BN or BED. Patients were excluded if they met criteria for clinical/subclinical anorexia nervosa (for which CBTgsh is not an evidence-based treatment). In Cycle 2, inclusion criteria for patients included: 1) 18 years or older, 2) US resident, 3) English-speaking, and 4) identified by their provider as experiencing binge eating with or without accompanying compensatory behaviors.

### Cycle 1 Recruitment

In Cycle 1, we recruited providers and patients separately to complete supervised usability testing of the online platform. Provider participants were recruited through social media posts, emails, partnerships with community mental health and psychology training clinics in the midwestern U.S., and mental health provider listservs (e.g., Missouri Eating Disorders Council, Academy for Eating Disorders). Recruitment materials highlighted the opportunity to participate in a study testing an online provider training and treatment platform for EDs. Providers interested in participating self-directed to an online eligibility screen. Providers who met inclusion criteria on the screen were given the opportunity to provide their contact information to be invited to participate in the study and subsequently completed a phone call with a study team member, during which the team member explained the study aims and procedures. Providers who were eligible and interested at this stage subsequently completed a baseline survey and provided informed consent to participate.

Patient participants in Cycle 1 were recruited through social media posts and emails. Recruitment materials for patients were directed to individuals with eating or body image concerns and highlighted the opportunity to participate in a study testing an online treatment platform for EDs. Patients interested in participating self-directed from recruitment materials to an online eligibility screen, which contained questions that screened for EDs. Patients meeting inclusion criteria on the screen were given the opportunity to provide their contact information to be invited to participate in the study and subsequently completed a phone call with a study team member, during which the team member explained the study aims and procedures. Eligible patients were subsequently sent a baseline survey, during which informed consent was obtained.

### Cycle 2 Recruitment

In Cycle 2, we recruited provider-patient dyads to complete unsupervised usability testing of the online platform. All procedures for recruiting providers in Cycle 2 were identical to those in Cycle 1.

During the initial phone call with a study team member, prospective providers in Cycle 2 were informed of the types of patients for whom the online CBTgsh platform would be a good fit (i.e., those with binge eating with or without accompanying compensatory behaviors) and verified whether they had or anticipated having a patient with a binge-type ED in the next two months. Providers were instructed to use their typical methods of assessing presence of eating pathology, an approach that has been used in other implementation research in EDs (21). After completing the baseline survey, enrolled providers were instructed to share general information about the study with their patients with a binge-type ED using IRB-approved information sheets about the study provided by the study team. Specifically, providers were instructed to inform patients that they were receiving remote training in an evidence-based treatment for EDs through a research study, and that if their patient was interested in participating, providers would begin guiding patients through CBTgsh material using the online platform to address their ED symptoms, as part of their usual care. Members of the study team provided support and reminders for providers making referrals to patients. Patients interested in participating self-directed to an online eligibility screen from the information sheets, and those who were eligible were sent a baseline survey on which they provided informed consent to participate. Patient participation in the study with their provider was voluntary, and data from providers whose patients were not interested in participating were not retained; that is, we only analyzed data from providers whose patients enrolled in the study.

### Online Platform Design

The CBTgsh web-based platform in this study (https://cbtforeatingdisorders.wustl.edu/) was developed, hosted, and maintained by an industry partner, 3C Institute. Participants were able to access the platform using any device with internet connection (e.g., computer, smartphone).

### Online Platform Content and Features

The CBTgsh content included in the online platform was based on the Overcoming Binge Eating, 2nd Edition self-help program for EDs (38). We created the original prototype online platform prior to usability testing based on prior implementations of CBTgsh (36, 39, 40) and consultation with experts involved in the original self-help program.

### Provider-Facing End

The provider-facing end of the online platform contained CBTgsh training materials, broken down into modules and delivered in numerous formats. Specifically, we created PDFs, videos, PowerPoints, and module summary sheets summarizing content in the Fairburn (38) self-help book. The training provided psychoeducation about EDs, a comprehensive description of the CBTgsh approach, guidelines for how to assess eating and body image problems in patients, and a session-by-session instructional walkthrough of how to deliver CBTgsh on a weekly basis with patients.

The provider-facing end also contained tools to support the implementation of CBTgsh with use of the platform, including session checklists with essential goals for each session and interactive sheets to take session notes on. Another key feature was that providers were given access to their patients’ real-time symptom self-monitoring data, which could be used to track patients’ progress.

### Patient-Facing End

The patient-facing end of the platform contained self-help content directly derived from Fairburn’s program (38), which included psychoeducation, goal-setting, and assignments broken down into modules. Specifically, the platform provided patients with chapters from the self-help book, as well as psychoeducational module cheat-sheets that we created to summarize the key learning points of each module and activity sheets (e.g., a shape-checking self-monitoring form). The platform also hosted digital self-monitoring logs, where patients could record their eating and ED symptoms; once entered, these data were immediately made visible to their providers. Screenshots of the provider- and patient-facing ends of the platform are included in the Supplementary Material.

### Procedures

Usability testing of the online CBTgsh platform was conducted over two cycles. After Cycle 1, refinements were made to the platform’s features and functionality based on feedback gathered from participants. All usability testing was conducted remotely to facilitate inclusion of participants across the United States. All procedures were overseen and approved by the Washington University in St. Louis Institutional Review Board.

### Cycle 1: Supervised Usability Testing

Upon completion of the baseline survey in Cycle 1, enrolled participants scheduled a 30-minute virtual testing session with a member of the research team. The testing session procedures were identical for both providers and patients. During the testing session, the research team member directed participants to the main features in the platform (i.e., instructions, dashboard, video, documents), a standard practice for assessing usability of an implementation strategy (41). During the walkthrough of the platform, participants were asked to use the “think aloud” strategy and voice aloud their thoughts and immediate reactions to the platform content (Jaspers et al., 2004). Following the testing session, participants participated in a 30-minute semi-structured qualitative interview to further assess their experience with the platform and feasibility. Participants were subsequently emailed a post-engagement survey, which contained quantitative measures of usability of the platform. Completion of all study activities in Cycle 1 took approximately one hour. Provider and patient participants were compensated with a $25 electronic Amazon gift card.

### Cycle 2: Unsupervised Usability Testing

Upon completion of the baseline survey in Cycle 2, enrolled providers were given access to the online platform (a unique account was created for each participant) and instructed to complete the CBTgsh training (which took about 3 hours to complete) via the online platform within one week. During this time, providers were also instructed to share information about the study with one of their patients for whom they believed this approach was a good fit (i.e., patient with binge eating with or without accompanying compensatory behaviors). After providers completed CBTgsh training and patients were consented and enrolled, providers were instructed to deliver CBTgsh to their patients using the online platform as part of usual care over a 3-week period. At the end of the testing period, semi-structured interviews were conducted with providers and patients separately to assess their experiences and feedback on the platform. Participants also completed post-engagement surveys which contained quantitative usability measures. Provider and patient participants were compensated with a $25 electronic Amazon gift card. Following completion of the study activities, providers and patients were able to continue using the platform if they wished.

## Measures

### Quantitative Data

At baseline, participants reported on demographic information, including race, ethnicity, sex, gender identity, sexual orientation, household income, and living region (including if they lived in a rural area).

Providers were also asked to indicate their profession (response options: 1) Psychiatrist; 2) Psychologist; 3) Therapist; 4) Counselor; 5) Social worker; 6) Mental health worker; 7) Other [please specify]); the highest degree they had received; whether they practiced in a rural area; and whether they practiced in a community mental health center.

The System Usability Scale (SUS) was used at post-engagement to evaluate the usability of the online platform. This measure contains 10 items, with response options ranging from strongly disagree (1) to strongly agree (5). Possible scores range from 0–100; overall scores above the established cutoff of 68 reflect “above average” usability. The SUS has been validated for use in small sample sizes (42, 43).

Participants also completed the Usefulness, Satisfaction, and Ease of Use (USE) Questionnaire at post-engagement. This 30-item measure assesses usefulness, ease of use, ease of learning, and satisfaction of users (44), with response options ranging from strongly disagree (1) to strongly agree (7). For each subscale, items were averaged to generate a score. Possible total scores range from 19–133.

The Stanford-Washington University Eating Disorder Screen (SWED) (45) was used on the eligibility screener in Cycle 1 to assess whether patients met criteria for clinical/subclinical BN or BED using the established criteria of endorsing 6 + binge eating episodes, 6 + vomiting episodes, and/or 6 + laxative/diuretic use episodes over the past 3 months. The SWED demonstrates good sensitivity and specificity for identifying DSM-5 ED diagnoses (45).

### Qualitative Feedback

Semi-structured interview questions solicited participant feedback on the individual platform components, the utility and design of the platform, and overall positive and negative experiences. The interview script for each cycle can be found in the Supplementary Material.

### Analytic Strategy

#### Quantitative Analysis

Descriptive statistics on participant characteristics and quantitative usability data were calculated using R version 4.1.3. Inferential statistics were not used given the small sample size.

#### Qualitative Analysis

##### Iterative Development

The initial version of the online CBTgsh platform was tested by Cycle 1 participants. Interviews with participants were transcribed and qualitative feedback was assessed and used to inform refinements to the platform before Cycle 2. For example, in response to participant feedback, we worked with 3C Institute to: 1) modify the data fields in the self-monitoring surveys (i.e., separate fields for place and time of logged eating event); 2) add language to self-monitoring surveys to instruct patients to save data before leaving the page; 3) improve organization of psychoeducational content; and 4) provide more training and treatment content on body image problems. Refinements were made based on feasibility and how frequently suggestions were made by participants. Some suggestions were not feasible given budget limitations.

##### Thematic Analysis

To examine provider and patient participants’ feedback on the online platform, the study team transcribed the recordings of the semi-structured qualitative feedback interviews from Cycle 1 and 2. We expected that our sample size (n = 18) sufficed for the purposes of qualitative analyses, given that sample sizes over 9 typically achieve coding saturation and sample sizes between 16–24 achieve meaning saturation (46, 47). We analyzed the transcripts using qualitative inductive thematic analysis with a realist lens focused on understanding the realities and experiences of the participants (48). Thematic analysis aims to identify repeating patterns and contexts of participant feedback and fit our analysis goals of assessing this feedback through an inductive, realist lens. In line with Braun & Clarke (48), we read the transcripts to understand participant feedback, created two separate codebooks for provider feedback and patient feedback, coded the transcripts (two independent coders coded each transcript), and defined and named themes.

##### Coding Procedures

During the coding process, each coder (n = 5) independently read and reviewed the transcripts and drafted preliminary codes. Then, the coding team came together and created an initial codebook, which was used to code a subset of the transcripts (1–2 transcripts per coder). After test coding using the initial codebook, all coders met several times to refine and finalize the codebook. Each transcript was coded by two independent coders using the finalized codebook. Coding discrepancies were identified and discussed by all coders until a consensus was reached. After completing coding, we used a bottom-up approach to: (1) group codes into subthemes based on their relationships within the transcripts, (2) group subthemes into themes, and (3) re-review transcripts and reevaluate themes and subthemes as needed. Finally, coders named and defined the themes and subthemes. The coding process and theme development were completed using the Dedoose software (49).

## Results

### Participant Characteristics

Nine mental health providers (M age = 41.8 ± 8.6, 88.9% female, 100% White and non-Hispanic) without expertise in CBTgsh participated in the study. Five providers (55.6%) practiced in community mental health centers and four (44.4%) practiced in rural areas. Table 1 describes characteristics of the provider participants.

Nine patients with probable EDs (77.8% female, 100% White and non-Hispanic) participated in the study. The mean age of patients in Cycle 1 was 53.8 (SD = 5.6); age of patients was not collected in Cycle 2. Table 2 describes patient participant characteristics.

### Quantitative Usability Data

See Table 3 for detailed usability data. In Cycle 1, providers reported a mean SUS score of 83.1 (SD = 12.6) and patients reported a mean score of 86.3 (SD = 18). These scores represent “good” and “excellent” usability, respectively. Providers reported a mean USE score of 111.5 (SD = 15.2) and patients reported a mean score of 124.5 (SD = 9.3).

In Cycle 2, providers reported a mean SUS score of 77.5 (SD = 10.2) and patients reported a mean score of 66.0 (SD = 18). These scores reflect “good” and “average” usability, respectively. Providers reported a mean USE score of 98.8 (SD = 25.1) and patients reported a mean score of 71.8 (SD = 30.8). Across the sample, usability scores declined between cycles but remained in the “good” or “acceptable” categories.

### Thematic Analysis

Thematic analysis revealed that data converged on two themes for providers and two themes for patients. Table 4 contains a summary of identified themes and subthemes.

Table 5 contains illustrative quotes from provider participants for each theme and subtheme. Themes are briefly described and illustrated below.

### Provider Theme 1: Applicability of the Program for Real-World Use

Providers’ feedback centered on implications for real-world use of the online platform. Specifically, their comments reflected their experiences using the platform and their thoughts about relevant factors for future use of the platform with providers and patients.

#### Barriers and Catalysts for Use

Providers commented on factors that facilitated and deterred them from using the platform during usability testing. In terms of catalysts of their use of the platform, they highlighted the ease of use of the platform, including overall easy navigation and ability to access resources and tools. They also cited specific features that they found useful as motivators for use of the platform. For instance, one provider commented, “I think if I had a patient for whom I really wanted to sort of track how they were eating and when they were eating and making sure they were eating regularly, then I would find it really helpful to be able to glance at [the self-monitoring logs] and just to have like a snapshot of how they’re doing” (P14). Providers also noted potential barriers to real-world use of the platform with patients, including scheduling challenges, limited time which may impede ability to use the platform, and seeing patients with limited tech-savviness.

#### Quality of Online Training Experience

Providers shared their impressions of the online platform’s CBTgsh training material, including the platform’s ability to provide training in a treatment approach that they did not have prior experience with. They praised specific characteristics of the training content, including the quantity, completeness, and organization of the content, as well as how relevant it seemed to themselves and patients. Several providers offered positive feedback on the various modalities of training content that was available in the platform (e.g., videos, PDFs). P10 commented, “I appreciated how the information was presented via video and then you had access to the actual slides and a checklist, like I feel like depending on what kind of a learner you have in front of you like you cover all of the different ways to absorb the information.” Providers found the training content to be informative and highlighted the introductory overview of CBTgsh as a standout component.

#### Utility of the Platform to Deliver Treatment

In addition to their experiences receiving training through the platform, providers discussed their impressions of how useful the platform was as an aid for delivering treatment for EDs. They mentioned the utility of specific features and tools for treatment delivery, such as session checklists, the food log, and the symptom tracker. One provider noted, “I really liked the checklists for the sessions and being able to kind of know I could go to that one tab and find everything I needed like, oh I’m running late, I have session one and I could pull it all up and print it all easily from there” (P11). Providers also offered their views on potential benefits to both providers (e.g., being able to review patients’ recent symptoms before session) and patients (e.g., being able to track their own progress in the platform) for providers using the platform to deliver CBTgsh.

### Provider Theme 2: Platform Structure and Function

Feedback from providers also centered on the ease of use of the platform, the platform’s aesthetics, and the functions of features and tools. Several providers highlighted that the platform was user-friendly, intuitive to use, and easy to navigate. One provider (P15) noted, “It’s certainly easy to log on, easy to find the main tabs, provider resources, patient resources, and to click along with the different modules.”

Table 6 contains illustrative quotes from patient participants for each theme and subtheme. Themes are briefly described and illustrated below.

### Patient Theme 1: Barriers and Facilitators to Engagement

Patients’ feedback on their experiences with the online platform focused on factors that facilitated and detracted from their use of the platform.

#### Platform Functionality

Patients discussed the functionality of the platform, including how well features and tools functioned, the ease of use of the platform, and the layout and aesthetics of the platform. Several patients highlighted challenges with functionality in the self-monitoring log and food log. For example, P08 noted, “Well, the only thing that I saw was like that technical issue, was, it just seems a little glitchy, like if I enter my data and then say I wanna go back to the dashboard and print it off, it will come up to be 0, there’s 0 entries in there. So I have to completely re-log in, then go to the dashboard, then it will load, then I can bring it up, choose to download it to a PDF, and print it off.” Patients commented on how such technical challenges detracted from the usability of the logs. Patients also highlighted that the platform was overall easy to navigate.

#### Patient-Specific Factors

Feedback from patients also discussed motivating and detracting factors for using the platform that were specific to the patients’ preferences and experiences (i.e., not related to platform functionality). They mentioned factors that may influence real-world use of the platform, such as level of comfort with technology. Patients provided suggestions for future iterations of the platform with improved accessibility for patients with lower comfort with technology. One patient stated, “[It would be helpful] having like a little instructional walkthrough, so that people don’t get too confused because there’s going to be people…that are not tech savvy at all that want to be able to use it” (P03). Patients also suggested simplification of platform features to improve accessibility: “That was the hardest part for me, [the amount of detail required]. Since I’ve gotten older, things need to be more simplified, I guess. When I’m younger I could multitask like everybody and their son” (P06).

### Patient Theme 2: Perceived Treatment Effects

Patients’ feedback also focused on the extent to which the platform was (or could have been) effective at addressing their ED symptoms.

#### Relevance to Therapeutic Goals

Patients provided feedback on the platform’s alignment with their goals for therapy. They specifically discussed the relevance of features and tools (e.g., the food log, symptom tracker, psychoeducation) and characteristics of platform content (e.g., quantity, personal relevance, clarify, format, completeness). Patients also reported their willingness to use the platform for further ED treatment, their willingness to recommend the platform to others, their perceived benefits of the platform (e.g., increased awareness, accountability), and the degree to which they felt that they could be honest while using the platform. P08 commented, “[What I liked about the platform was] the accountability of actually having to log information in, to have to self-assess what you would characterize as a binge, knowing this is going to be looked at by somebody else. So you’re under a microscope, you know, so it’s time to get real, time to be honest with yourself, and that really helped me. That really helps me. Just seeing it in black and white. I’d say the accountability factor was huge and that’s what I was really afraid of losing, to be honest.” In addition to accountability, patients mentioned that the content (e.g., psychoeducation) and tools (e.g., symptom tracking) in the platform allowed them to develop more awareness of their symptoms: “I love the idea that you could actually write your response to gauge how you’re feeling at the time you’re eating. I love that. That was the absolute best part. Because it actually made me think a lot about things I never thought of before where my food was concerned. When I would sit down to read, I felt almost like somebody understood me while reading these articles. When I was writing, when I filled out the food journals, [I would think], ‘Hold up, is this why I’m eating this? Am I hungry, or…?’ I felt like somebody finally got it.” (P06)

#### Patient-Provider Communication

Patients commented on how they used the platform to communicate with their treatment provider. They reflected that the self-monitoring logs allowed them to send timely reports on their symptoms to their providers. Patients also highlighted how the platform’s tools allowed for private forms of communication with their providers, which facilitated honest disclosure. One patient stated, “[I think the self-monitoring surveys would be helpful for providers to assess patients’ progress] because some people aren’t comfortable talking to their providers about it even though that’s what they’re there for, so sometimes it might be easier just to do this and submit that information and then they can possibly try to do some diagnosing with what they’ve submitted” (P01).

## Discussion

This study employed user-centered design to develop a prototype online platform for disseminating training for mental health providers in CBTgsh and supporting its implementation. We conducted two iterative cycles of usability testing with mental health providers without prior training in CBTgsh and adult patients with EDs. To our knowledge, this was the first “all-in-one” online platform developed to support both scalable training of providers in an evidence-based treatment and intervention delivery for EDs.

In Cycle 1 of usability testing, we recruited providers and patients separately to complete brief platform testing sessions. In Cycle 2, we recruited provider-patient dyads; providers completed training using the platform and subsequently delivered CBTgsh to a patient for three weeks. Despite the fact that refinements based on Cycle 1 feedback were made to the platform prior to Cycle 2, usability scores decreased between cycles for both providers and patients. Because of the nature of unsupervised testing, it is plausible that the lower usability scores in Cycle 2 were driven by unique needs of providers and patients during routine clinical care. Indeed, thematic analysis highlighted that patients experienced challenges with the functionality of some platform tools (e.g., self-monitoring logs) in routine practice. Patients also discussed personal factors (e.g., lack of comfort with technology) that contributed to poorer usability. These findings reflect the importance of collecting usability data under real-world conditions to inform refinements that serve users’ needs, which has been called for by many researchers in the digital mental health implementation field (50, 51). Despite the decrease in usability, scores remained in the average to good range across both cycles. Qualitative feedback suggested that providers and patients saw utility in the platform’s training and treatment capabilities; these data suggest that further refining the platform’s functionality and accessibility could improve its usability.

Thematic analysis of participant feedback revealed provider themes of applicability of the platform for real-world use and platform structure and function. On the whole, providers reported high ease of use of the platform. They found the training material to be informative, organized, and well-formatted. Providers had positive impressions of the platform’s treatment implementation support tools, such as the session checklists and patient self-monitoring surveys, due to their ability to help them prepare for sessions and track their patients’ progress.

Patients’ qualitative feedback centered on barriers and facilitators to platform engagement and perceived treatment effects. Patients reported considerably lower ease of use of the platform relative to providers, citing challenges with navigation and issues with functionality of self-monitoring logs. They cited low comfort with technological tools as a barrier to using the platform and provided feedback for improving accessibility for less tech-savvy patients. However, patients generally found the platform’s treatment content and tools to align with their treatment goals and reported benefits such as increased accountability and awareness of their symptoms following use of the platform. Patients also highlighted the platform’s ability to facilitate discrete and timely patient-provider communication. This feedback suggests that patients found the platform to have high potential to address their ED symptoms, and that further refining the platform to improve functionality and accessibility could improve its effectiveness.

Strengths of this study included the use of a rigorous thematic analysis protocol, in line with Braun & Clark (48), and the diversity of the mental health providers in terms of practice setting. Another strength was the use of user-centered design with target users (i.e., mental health providers without expertise in CBTgsh and patients with EDs) under real-world conditions, which will enhance scale-up of the online platform (35). Limitations included lack of diversity in the sample in terms of race, ethnicity, gender identity, and sex, as well as lack of data on age of patients in Cycle 2. Another limitation was that we were not able to make all suggested refinements between cycles due to limited capabilities by the budget for our pilot study, which may have impacted usability. Importantly, the providers enrolled in this study were largely highly trained clinicians (without expertise in CBTgsh) and had to have access to patients with EDs to participate. Because CBTgsh is intended to be delivered by a wide variety of people with minimal training (i.e., no experience with treating EDs or with CBTgsh) to maximize scalability, data from this study may not generalize as well to its intended population of providers. Although usability data from the present sample is highly valuable, future research evaluating the provision of online training to non-specialist providers is critically needed to uncover CBTgsh’s true potential as a scalable solution for addressing the ED research-practice gap.

## Conclusions

Taken together, findings from this study demonstrate preliminary feasibility and acceptability of the online platform. Results indicate areas for improvement to increase usability of the platform, yet the approach was largely received well by both providers and patients. Data collected in this study will inform further refinements to the online platform, and the platform’s effects on provider training outcomes and patient ED symptom outcomes will be evaluated in an open pilot trial. Given the wide treatment gap for EDs (6, 7) and barriers to dissemination of evidence-based treatments (14), the online platform represents a scalable solution that could improve access to evidence-based care for EDs.

## Figures and Tables

**Table 1:** Provider participant characteristics (N = 9).

**Age, M (SD)**	**41.8 (8.6)**				
**Gender Identity, N (%)**	**Female**	**Male**	**Non-binary**		
	8 (88.9%)	0 (0.0%)	1 (11.1%)		
**Sex, N (%)**	**Female**	**Male**			
	9 (100.0%)	0 (0.0%)			
**Race, N (%)**	**White**	**Black/African American**	**Asian**	**American Indian**	**Native Hawaiian**
	9 (100.0%)	0 (0.0%)	0 (0.0%)	0 (0.0%)	0 (0.0%)
**Ethnicity, N (%)**	**Hispanic/Latino**	**Non-Hispanic/Latino**			
	0 (0.0%)	9 (100.0%)			
**Sexual Orientation, N (%)**	**Heterosexual**	**Bisexual**	**Queer**	**Gay/Lesbian**	**Other/self-identified**
	7 (77.8%)	1 (11.1%)	1 (11.1%)	0 (0.0%)	0 (0.0%)
**Income, N (%)**	**$40k-59.9k**	**$60k-79.9k**	**$80k-99.9k**	**$100k-149.9k**	**$150k+**
	1 (11.1%)	1 (11.1%)	2 (22.2%)	2 (22.2%)	2 (22.2%)
**Profession, N (%)**	**Therapist**	**Psychologist**	**Psychiatrist**		
	5 (55.6%)	3 (33.3%)	1 (11.1%)		
**Highest Degree Received, N (%)**	**Master’s**	**Doctorate**			
	5 (55.6%)	4 (44.4%)			
**Geographic Area of Practice, N (%)**	**Rural**	**Non-rural**			
	4 (44.4%)	5 (55.6%)			
**Community Mental Health Center, N (%)**	**Yes**	**No**			
	5 (55.6%)	4 (44.4%)			

**Table 2:** Patient participant characteristics (N = 9).

**Gender Identity, N (%)**	**Female**	**Male**		**Non-binary**				
	7 (77.8%)	1 (11.1%)		1 (11.1%)				
**Sex, N (%)**	**Female**	**Male**						
	8 (88.9%)	1 (11.1%)						
**Race, N (%)**	**White**	**Black/African American**		**Asian**	**American Indian**	**Native Hawaiian**		
	9 (100.0%)	0 (0.0%)		0 (0.0%)	0 (0.0%)	0 (0.0%)		
**Ethnicity, N (%)**	**Hispanic/Latino**	**Non-Hispanic/Latino**						
	0 (0.0%)	9 (100.0%)						
**Sexual Orientation, N (%)**	**Heterosexual**	**Bisexual**		**Queer**	**Gay/Lesbian**	**Other/Self-identified**		
	8 (88.9%)		1 (11.1%)	1 (11.1%)	1 (11.1%)	0 (0.0%)		
**Income, N (%)**	**<$20k**		**$20k-39.9k**	**$40k-59.9k**	**$60k-79.9k**	**$80k-99.9k**	**$100k-149.9k**	**$150k+**
	2 (22.2%)		1 (11.1%)	0 (0.0%)	1 (11.1%)	0 (0.0%)	4 (44.4%)	1 (11.1%)
**Geographic Area, N (%)**	**Rural**		**Non-rural**					
	4 (44.4%)		5 (55.6%)					

**Note**. Percentages for the sexual orientation categories exceed 100% because participants were able to select all categories that applied.

**Table 3:** Usability scale scores.

	Cycle 1		Cycle 2	
	Providers	Patients	Providers	Patients
**SUS Total**	83.1 (12.6)	86.3 (1.8)	77.5 (10.2)	66.0 (15.9)
**USE Total**	111.5 (15.2)	124.5 (9.3)	98.8 (25.1)	71.8 (30.8)
**USE Usefulness**	6.0 (0.4)	6.6 (0.2)	4.6 (2.1)	3.4 (2.3)
**USE Ease of Use**	6.0 (0.0)	6.5 (0.4)	5.6 (0.5)	4.1 (1.5)
**USE Ease of Learning**	6.2 (0.2)	6.4 (0.1)	6.0 (0.9)	4.5 (2.1)
**USE Satisfaction**	5.7 (0.3)	6.7 (0.2)	5.0 (1.7)	3.4 (2.3)

*Note*. SUS Total ranged from 0–11. USE Total ranged from 19–133. USE subscales ranged from 1–7.

**Table 4:** Main themes and subthemes of participant feedback.

Provider Theme 1	Provider Theme 2	Patient Theme 1	Patient Theme 2
Applicability of Program for Real-World Use	Platform Structure and Function	Barriers and Facilitators to Engagement	Perceived Treatment Effects
Barriers and Catalysts for Use		Platform Functionality	Relevance to Therapeutic Goals
Quality of Online Training Experience		Patient-Specific Factors	Patient-Provider Communication
Utility of the Platform to Deliver Treatment			

**Table 5:** Themes, descriptions, and feedback from providers. .

Themes	Description	Illustrative Quotes (P#)
Platform Structure and Function		
N/A	Ease of use and aesthetics of platform features and tools.	“It’s certainly easy to log on, easy to find the main tabs, provider resources, patient resources, and to click along with the different modules.” (P15) “So I would say it was not that easy. I mean it’s easier than not having it, but you know, it was hard to say. It was easy to use it, but I got caught up in these other things, like how long was this? How many things really are there that I have to watch? And this seems a lot, you know.” (P16) “It was pretty simple for me to be able to log in and look at her food logs and have a conversation with her as I was looking at the log. So from that perspective for me, it was pretty simple.” (P17) “I mean [the platform] was very user friendly, intuitive. I didn’t… you know again, I wasn’t the one having to enter anything into there. So you know, for me to be able to look at it in real time while I was on the phone with the patient was very helpful.” (P17) “For me, I find it easy. You know, once I oriented myself to it, and really figured out the system of where everything was located in the steps, and all of that, I’ve I found it very easy to use and easy to be able to see what my patient has done on it.” (P18) “Oh it was definitely user-friendly, it was, with the tabs and stuff you could find your way around pretty simple which was good.” (P11)
Applicability of Program for Real-World Use		
Barriers and Catalysts for Use	What motivated or detracted providers from using the platform.	“I think if I had a patient for whom I really wanted to sort of track how they were eating and when they were eating and making sure they were eating regularly, then I would find it really helpful to be able to glance at that and just to have like a snapshot of how they’re doing.” (P14) “I felt like, again, even with my limited experience, I didn’t fully appreciate or grasp the provider resources until after I looked at the patient resources. So that might have been useful to have been kind of instructed from the beginning.” (P14) “I have other patients I’m doing this treatment with who are not tech savvy, who I haven’t even considered like explaining the study to them, because I think it would just explode and it’s been hard enough getting her, you know I kind of have taught her how to use the excel sheet, and that type of thing. So I wouldn’t, I wouldn’t recommend it, it sort of needs a very specific demographic, I think, of a, you know, somebody who’s tech savvy, knowledgeable, pretty young, you know, in terms of their ability to access it. Cause I think other patients of mine would just find the concept of it a little bit overwhelming, though I really like it. I like everything that’s there. I just don’t know if it’s translatable to every patient who does this treatment, if that makes sense.. (P18) “My experience wasn’t typical because my patient got upset at how much binge-eating was mentioned right away. I didn’t know her. She had gotten an intake, and then I wanted to do CBT. She had agreed, and her intake showed that she did engage in binge eating. It wasn’t that frequent, but she did. And then, as it went on, she just got very triggered by it, but she’s a patient who’s triggered by lots of things, and that is just sort of part of what is going on.” (P16) “I liked how I could see the information in multiple ways through video, through checklists, through your slides. And I imagine that like, if I really was knowledgeable about what was coming in terms of each session that it would be really helpful to be able to filter by the type, like pdf versus video, so that like I could really quickly find what I needed and even be able, as a provider, to like, I imagine if I had the time that I would like to sit down and like watch all the videos from start to end and like review all the slides from start to end and that’s a really nice way to filter in order to do that. So yeah I liked that part about the provider end.” (P10) “I felt like, again, even with my limited experience, I didn’t fully appreciate or grasp the provider resources until after I looked at the patient resources. So that might have been useful to have been kind of instructed from the beginning. “Please. First look at the patient resources. That will be a helpful context for you to get a sense of how to use the provider resources.” (P15)
Quality of Online Training Experience	Provider feedback on the content and relevance of the platform’s online eating disorder training.	“So overall, I found it really informative and useful. I really enjoyed the content. Like, I enjoyed the videos, the PowerPoint slides, and then the chapters. I actually downloaded some of the chapters for just me to read and reference. So I thought it was really, really helpful.” (P14) “Like I said, I would’ve loved to, for the person that was talking through the orientation, like the initial session, I would’ve for the video to be a little bit bigger to like see her face but that’s just because I like to watch people talk, and I’m not as much of like a reader, so maybe enlarging that video a little bit, and yeah, I loved it. I loved how she was talking through all of the slides and it was offered in multiple ways for multiple learners.” (P10) “[My experience using the platform] was good. I appreciated how the information was presented via video and then you had access to the actual slides and a checklist, like I feel like depending on what kind of a learner you have in front of you like you cover all of the different ways to absorb the information.” (P10) “[In terms of resource improvement], you can’t give me more time, so I don’t I wanna be unreasonable. I mean, I know again the patient gave me feedback about the reading, and that some of the reading would refer to different parts of the book that she didn’t have access to, that she would have liked to be able to look at and then maybe yeah. I mean, that could be my feedback, too, is that would be more information that I could have access to, to give a little more depth to some of the information. But, you know, I’m really struggling to be critical. It was very helpful.” (P17) “I actually liked the person who was doing the training videos, normally I want to go to sleep during training videos, but she actually kept me entertained, so that’s pretty good.” (P11) “I really enjoyed [the training]. I really liked how organized it was, and I really liked some of the sort of key takeaways. So, for example, one of the things that really stood out to me was like comparison making. And I really like the sort of idea of instead of comparing yourself to a biased sample, comparing yourself to everybody in a systematic way, every third person. So I liked how it was a combination of sort of just like background and educational, but then also just like takeaway tips and things to try.” (P14)
Utility of the Platform to Deliver Treatment	Provider thoughts on how useful the platform was.	“The checklist was helpful. I have that here in front of me, and it was even more helpful again, as I mentioned, after I had looked at the patient resources, the provider resources were more helpful afterward.” (P15) “I really liked the checklists for the sessions and being able to kind of know I could go to that one tab and find everything I needed like, oh I’m running late, I have session one and I could pull it all up and print it all easily from there.” (P11) “For the food checker, where there were just, you know, fifteen options for a single entry, so there was just a lot of scrolling that would need to be done, which I could imagine could get a little bit much if you’re like trying to track like which encounters or like what interaction is this, what, I forget, time, what word you used, but like what time point is this.” (P10) “I think [the self-monitoring surveys would be helpful for patients to keep track of their own progress…to kind of have that accountability and empowerment and awareness. Each of those can be enhanced with the intentional monitoring of what we do that might otherwise be mindless.” (P15) “From a clinician perspective, the self-monitoring form is great, and I really really like, here’s a huge thing of the platform that I like, that as the clinician, if you want to, you can sort of review throughout the week how your patient is doing, or what’s going on. You know, it’s a place where I can go check in on. I don’t need to just be with the patient and to talk about. So it means that before I meet with her, I can review. So that shared aspect of it I think is fantastic. That’s the reason why this platform should exist. Cause it can be accessible to both the clinician and the patient.” (P18) “I definitely think I could gain a better understanding of eating disorders and how to treat them from using this platform because. I’ve been doing this for a long time, but when I very first started out, I think it would’ve been extremely helpful to have something kind of guiding so that you don’t feel like you’re questioning yourself: am I moving in the right direction, am I staying on track, are we doing this as brief as a format as possible, um, for the client so that they’re getting the help they need, and so I think that that would be helpful for anybody at any level of experience to just check in with yourself and be able to use this to help keep you moving, keep you on track, and keep you assessing what you need to assess at each step of the way.” (P13)

**Table 6:** Themes, descriptions, and feedback from patients.

Themes	Description	Illustrative Quotes (P#)
Barriers and Facilitators to Engagement		
Platform Functionality	Ease of use and layout of platform features and tools.	“When I when I first went into it, I had a really hard time navigating it, so I felt like um. Like if I were going to use this app on a daily basis then it would have been a much simpler way to get into it. I felt like when I went into it I was confused at where I was to where I was supposed to be. Do you understand what I’m saying? And I would think every time, is this where I went last time? And that’s a lot of what I did was, did I do this last time?” (P06) “Dashboard was fine, super easy to navigate. You know if I went to get to the log or to the lessons, like you know, a couple of clicks and you are there.” (P05) “…I do a lot on my iPad, and so I found that if I start like logging in my meals, you know, in real time, and then I will leave that application to open another application. When I come back to it, it reboots and erases everything. So that was kind of discouraging.” (P08) “Well, one thing that would be kind of cool is where it says ‘context and comments.’, you know, we don’t really have much room…I print my logs out for my own personal reference, and there’s only so much space after which it will cut off, so,…it might be kind of cool, if it could somehow incorporate a link for maybe a journal, like where you could like journal some thoughts and things if the person wanted to… There’s just not a whole lot of room anywhere that I saw that you could really type in thoughts and feelings or things like that” (P08) “The problem with the self-monitoring approach, the report approach is that there’s no way to see all of the days together, and as a user, there’s no real way for me to track as I go. I either need to track everything as I go using a Google sheet, which is what I did…So I didn’t find that the dashboard [was] really functional, given the whole purpose of monitoring, and then being able to communicate with the provider through the platform about what you monitored.” (P09) “Well, the only thing that I saw was like that technical issue, was, it’s just seems a little glitchy, like if I enter my data and then sa I wanna go back to the dashboard and print it off, it will come up to be 0, there’s 0 entries in there. So I have to completely re-log in, then go to the dashboard, then it will load, then I can bring it up, choose to download it to a PDF, and print it off. It just seems a little glitchy, that you have to log back in each time.” (P08)
Patient-Specific Factors	What motivated or detracted patients from using the platform for reasons aside from how the platform functioned.	“I had really just like I said, a hard time, like getting actually getting to where I need to be on the food journal. That was a lot of my problem. But, everything it’s like. Maybe I didn’t take the time to get to know it like I should have. Or maybe I was just so frustrated that, I mean, there was 100 things going on with me at the time.” (P06) “I just, that was the only thing I had technical difficulties. I think if I would have had somebody in front of me. You know, like sitting down. Explain to me. I work a lot better that way because when I get confused, I kind of end up in a circle. And with me, that’s my own fault. I can’t, I can’t help that.” (P06) “Like I said, [it would be helpful] having like a little instructional walkthrough, so that people don’t get too confused because there’s going to be people…that are not tech savvy at all that want to be able to use it” (P03) “I did [get frustrated] because I really didn’t ask for a lot of help. I did try to just do that on my own and then when I get frustrated, I would just, you know, I don’t know.” (P06) “That was the hardest part for me, [the amount of detail required]. It, things really since I’ve gotten older, need to be more simplified, I guess. When I’m younger I could multitask like everybody and their son.” (P06) “It was user friendly. I’ll say that, cause Like I said, I have only limited computer skills. So it was easy to figure out, easy to follow.” (P08)
Perceived Treatment Effects		
Relevance to Therapeutic Goals	Patient feedback on platform alignment with their aims in therapy.	“[What I liked about the platform was] the accountability of actually having to log information in, to have to self- assess what you would characterize as a binge, knowing this is going to be looked at by somebody else. So you’re under a microscope, you know, so it’s time to get real, time to be honest with yourself, and that really helped me. That really helps me. Just seeing it in black and white. I’d say the accountability factor was huge and that’s what I was really afraid of losing, to be honest.” (P08) “I love the idea that you could actually write your response to, like, OK gauge how you’re feeling at the time you’re eating. I love that. That was the absolute best part. And I actually would love to see more of that. That was the best part of it all and I loved the articles, being able to read those, that I enjoyed. Because it actually made me think a lot about things I never thought of before where my food was concerned. When I would sit down to read, I felt almost like somebody understood me while reading these articles. When I was writing, when I filled out the food journals, [I would think], ‘Hold up, is this why I’m eating this? Am I hungry, or.?’ I felt like somebody finally got it.” (P06) “I mean, the platform was good, just the content wasn’t really what I was looking for. Like in the binge eating and the vomiting or stuff like that. We were just more focused on body image and stuff like that. I think body image had maybe one chapter at the end.” (P05) “I’m really starting to [have a better understanding of my eating-related symptoms after using this platform]. Like I said, I am excited to get into the reading material, step 3 especially, and step 4, problem-solving. I’m just excited to keep going. Because I think it’s really not something that anyone else has ever been able to give me answers to my questions of: Why, why is this such a hurdle for me? Why, why do I have these problems with eating? Why, why is this happening? What throws me into a spin? You know. And some of those things I have to answer for myself, but you have to ask the right questions, if that makes any sense. It’s very thought-provoking, let me say that, to do the self-examination. It’s been a good process.” (P08) “I think [my symptoms could improve while using the platform]. Because sometimes we’re not aware of how much we’re eating, we’re just going through the motions, like being in the pandemic, it was hard sitting around the house and you’re eating because your bored or you’re eating because you’re frustrated or you’re eating because you’re overwhelmed, anxious, whatever. And I think that would be very helpful.” (P03) “[I think the self-monitoring surveys could] probably be helpful, but again my point earlier, I guess I don’t like the repetitiveness; I like to get to the point for a solution for me. Maybe for some people it helps.” (P04)
Patient-Provider Communication	Patient feedback on how they used the platform to communicate with their treatment provider.	“I think when you’re putting a log in [the self-monitoring surveys] and there’s certain notes that you can put in, you know sometimes like you don’t feel comfortable talking about something, but you can type something into the notes section that your therapist can look at and say, ‘OK, well, maybe here’s like a trigger, or here’s some background that’s important to as why this happened.” (P05) “Like, with my situation, my doctors put me on metformin because my A1C is too high, I’m not diabetic but I’m prediabetic, and I think having that information not just written down but in data [in the self-monitoring surveys] and you can show him, I can show him what I’ve been eating, how much I’ve been eating, and then maybe get ahold of, get on top of everything from there.” (P03) “I would imagine, you know, providers have to enter stuff in their electronic health records. Like this allows for, you know, digital data creation is great. So then is there a way for someone for a patient who’s less comfortable with that to also benefit from the platform, and you know, strengthen their engagement with the provider, even if they are less comfortable with navigating digital spaces.” (P09) “I think [I would consider using the platform for continued treatment], but I kind of want to understand why [the platform] is better than just sharing the documents via the messaging that I already have with my doctor.” (P07) “[I think the self-monitoring surveys would be helpful for providers to assess patients’ progress] because some people aren’t comfortable talking to their providers about it even though that’s what they’re there for, so sometimes it might be easier just to do this and submit that information and then they can possibly try to do some diagnosing with what they’ve submitted.” (P01) “So right now, I think what’s most helpful about the treatment is the kind of structure of mindfulness, like creating this process and structure of self-monitoring and reflection, and then not being on my own with it, being able to engage with a therapist for me has been particularly helpful. So again I can see the platform being a really great bridge, and really facilitating that benefit well with the kind of evolution, or something to that effect based on what you folks do.” (P09)

## Data Availability

The datasets used and/or analyzed in the current study are available from the corresponding author on reasonable request.

## Abbreviations

CBTgsh: cognitive-behavioral therapy guided self-help
EDE-Q: Eating Disorder Examination-Questionnaire
EDs: eating disorders
SWED: Stanford-Washington Eating Disorder Screen
